# Risk factors for psychiatric readmission among inpatients with major depressive disorder: A patient-chart based study

**DOI:** 10.1192/j.eurpsy.2023.1776

**Published:** 2023-07-19

**Authors:** H. Al-Haboobi, M. Ioannou, M. Ben Saleh, A. E. Bakken Wold, S. Berg, S. Patraskovic, Z. Szabó, S. Steingrímsson

**Affiliations:** 1 Institute of Neuroscience and Physiology, University of Gothenburg, Sahlgrenska Academy; 2 Psykiatri Affektiva, Department of Psychiatry, Sahlgrenska University Hospital, Gothenburg, Sweden

## Abstract

**Introduction:**

Major depressive disorder (MDD) is a common and severe mental disorder. Although inpatient care may be needed in some cases, little is known on which factors are associated with risk for readmission.

**Objectives:**

To identify risk factors associated with an increased risk of readmission within 90 days, after being discharged from psychiatric inpatient care for depression.

**Methods:**

A medical record review is ongoing based on consecutive inpatients admitted in 2019-2021 at Sahlgrenska University Hospital, in Sweden. Inclusion criteria are MDD-diagnosis, admission > 7 days, no admission during the past half-year. Exclusion criteria are blocked medical record, patients who expired within 90 days after discharge. Time to first readmission for discharged patients was examined within 90 days. Clinical and sociodemographic characteristics were compared between readmitted and no-readmitted patients.

**Results:**

To date, 446 cases have been included with a readmission rate of 19.5%. In a subgroup of 182 patients (admitted between April 2020 and March 2021), psychotic subtype of depression seems to be protective to re-admission (p < .003) while comorbid eating (p < .017) and neurodevelopmental disorder (p < .029) seem to be associated with high risk. At the congress, results from the whole cohort will be presented.

**Conclusions:**

Medical record reviews can give good clinically relevant data for prediction of readmission. Comorbidities and depression subtypes may affect the risk for readmission.

**Disclosure of Interest:**

None Declared

